# Multistate Reaction Coordinate Model for Charge and Energy Transfer Dynamics in the Condensed Phase

**DOI:** 10.1021/acs.jctc.3c00770

**Published:** 2023-10-10

**Authors:** Zengkui Liu, Haorui Hu, Xiang Sun

**Affiliations:** † Division of Arts and Sciences, NYU Shanghai, 567 West Yangsi Road, Shanghai, 200124, China; ‡ NYU-ECNU Center for Computational Chemistry at NYU Shanghai, 3663 Zhongshan Road North, Shanghai, 200062, China; ¶ Department of Chemistry, New York University, New York, New York, 10003, United States; § Shanghai Frontiers Science Center of Artificial Intelligence and Deep Learning, NYU Shanghai, 567 West Yangsi Road, Shanghai, 200124, China

## Abstract

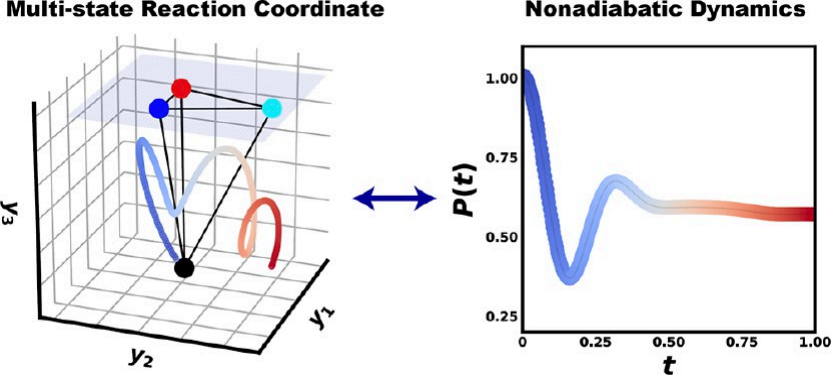

Constructing multistate model Hamiltonians from all-atom electronic structure calculations and molecular dynamics simulations is crucial for understanding charge and energy transfer dynamics in complex condensed phases. The most popular two-level system model is the spin-boson Hamiltonian, where the nuclear degrees of freedom are represented as shifted normal modes. Recently, we proposed the general multistate nontrivial extension of the spin-boson model, i.e., the multistate harmonic (MSH) model, which is constructed by extending the spatial dimensions of each nuclear mode so as to satisfy the all-atom reorganization energy restrictions for all pairs of electronic states. In this work, we propose the multistate reaction coordinate (MRC) model with a primary reaction coordinate and secondary bath modes as in the Caldeira-Leggett form but in extended spatial dimensions. The MRC model is proven to be equivalent to the MSH model and offers an intuitive physical picture of the nuclear-electronic feedback in nonadiabatic processes such as the inherent trajectory of the reaction coordinate. The reaction coordinate is represented in extended dimensions, carrying the entire reorganization energies and bilinearly coupled to the secondary bath modes. We demonstrate the MRC model construction for photoinduced charge transfer in an organic photovoltaic caroteniod-porphyrin-C_60_ molecular triad dissolved in tetrahydrofuran as well as excitation energy transfer in a photosynthetic light-harvesting Fenna-Matthews-Olson complex. The MRC model provides an effective and robust platform for investigating quantum dissipative dynamics in complex condensed-phase systems since it allows a consistent description of realistic spectral density, state-dependent system-bath couplings, and heterogeneous environments due to static disorder in reorganization energies.

## 1 Introduction

Photoinduced energy conversion inevitably involves quantum transitions between multiple electronic states, for instance, photoinduced charge transfer (CT) and excitation energy transfer (EET) in photovoltaic cells, organic electronics, and photosynthetic light-harvesting complexes.^1−16^ Theoretical calculations for such processes can help to elucidate the key factors that contribute to the efficiency and pathways of energy conversion. The microscopic mechanisms of charge and energy transport dynamics has a nonadiabatic nature, where the Born–Oppenheimer approximation of separating electronic and nuclear degrees of freedom (DOF) breaks down.^17−20^ Theoretical understanding of such processes requires suitable dynamic theories as well as effective Hamiltonians.

In recent years, many quantum dynamical methods were developed to simulate the “numerically” exact charge and energy transfer dynamics, including real-time path integral approaches,^21,22^ hierarchical equations of motion (HEOM),^23,24^ and wave function-based multiconfiguration time-dependent Hartree (MCTDH),^25−27^ time-dependent density matrix renormalization group (TD-DMRG),^28−30^ and tensor-train thermofield dynamics (TT-TFD),^31,32^ to name a few. These methods could only treat simple low-dimensional systems such as single molecules or model Hamiltonians because the computational cost increases drastically with the system size. On the other hand, approximate nonadiabatic dynamical methods have found a much larger application scope ranging from complex molecular to condensed-phase systems, such as mixed quantum-classical,^17−20^ quasiclassical,^33−36^ and semiclassical^37−39^ methods.

For condensed-phase systems, it is desirable to simulate the nonadiabatic dynamics with all-atom details, but the prohibitively large dimensionality makes this task difficult. Using an effective model Hamiltonian offers a very attainable and straightforward computational alternative to the problem. A common strategy is to propose a form of model Hamiltonian whose parameters are either assumed or deduced from experiments or calculations.^40−47^ For example, one of the best approaches is to construct effective models by mapping from the all-atom anharmonic Hamiltonian with the help of electronic structure calculations and molecular dynamics simulations.^44−47^

The most popular model for charge and energy transfer in a two-level system is the spin-boson Hamiltonian, which has been applied to study many quantum dissipative dynamics.^46−49^ In spin-boson Hamiltonian, the nuclear DOF are represented as a collection of shifted normal modes, which are bilinearly coupled to the electronic DOF. The harmonic normal mode approximation works rather well in the condensed phases due to a large number of molecules and the central limit theorem in contrast to the situation in a single molecule where some relevant torsional motion may not be well described by a harmonic potential. Denoting the two electronic states as the donor state |*D*⟩ and the acceptor state |*A*⟩, we can express the spin-boson Hamiltonian as
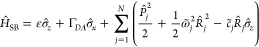
1where  and  are the electronic Pauli matrices; Γ_*DA*_ is the electronic coupling; −Δ*E* = 2ε = *ℏω*_*DA*_ is the donor-to-acceptor reaction free energy. In addition,  are the mass-weighted coordinates, momenta, and frequencies of the *N* nuclear normal modes, respectively, and  are the electronic-nuclear coupling coefficients. The parameter values for  can be determined by the spectral density defined as
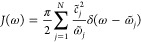
2

The spectral density sometimes is assumed to be certain analytical forms such as Ohmic and Debye types^40,41,49,50^ or can be obtained from realistic all-atom simulations using its direct relation with the time correlation function (TCF) of the energy gap between two electronic states.^44−47^ The reorganization energy between the two states is proportional to the variance of their energy gap and also related to the spectral density integral:
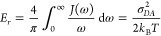
3where  and *U*_*DA*_ = *V*_*D*_ – *V*_*A*_ is the donor–acceptor energy gap.

**Figure 1:**
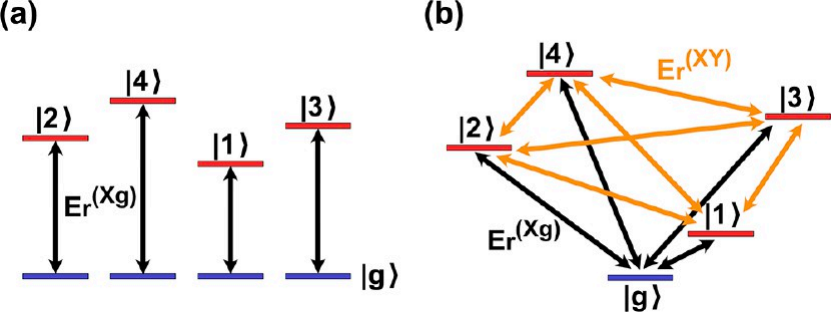
Comparison of traditional isolated bath models (a) such as the Frenkel exciton model with reorganization energy restrictions only between excited states and their own local bath ground states (black) versus our multistate harmonic (MSH) or multistate reaction coordinate (MRC) models (b) with reorganization energy restrictions between all possible pairs of states including excited state-excited state correlations (orange).

However, when one goes beyond the two-state scenario, proposing an effective model Hamiltonian that is consistent with all-atom Hamiltonian becomes more challenging because the number of reorganization energies is not equal to the number of electronic states.^51,52^ In a general *F*-state system, the fundamental problem is that there are *C*_*F*_^2^ = *F*(*F* – 1)/2 pairs of energy gaps and thus *F*(*F* – 1)/2 reorganization energy restrictions, which is more than the number of states. In the traditional isolated bath models such as the Frenkel exciton model, as shown in Figure 1(a), each excited state is connected to its own bath characterized by the reorganization energy between this excited state and the ground state (the local bath), and there is no communication between the baths belonging to different excited states. Essentially, the isolated bath model could use only *F* excited-/ground-state reorganization energy restrictions from all-atom simulations.

Recently, we proposed a consistent way to incorporate all reorganization energies in the multistate harmonic (MSH) Hamiltonian.^53^ The idea is that one needs to extend the spatial dimension of each normal mode from 1 to *F* – 1 in order to simultaneously satisfy the *F*(*F* – 1)/2 reorganization energy restrictions from all-atom simulations. As shown in Figure 1(b), the ground state and all excited states are treated on an equal footing, and the communications between all pairs of states are accounted for in terms of the reorganization energies. Effectively, the locations of the potential energy surface (PES) minima of all *F* states can be represented by vertices of a polyhedron in *F* – 1-dimensional space, whose edge lengths are proportional to the square roots of the corresponding reorganization energies.^53^ Incorporating the reorganization energy restrictions between excited states is important. For example, it has been demonstrated that the MSH model provides accurate nonadiabatic dynamics compared with all-atom nonadiabatic semiclassical dynamics in a prototypical photoinduced CT process involving a carotenoid-porphyrin-C_60_ (CPC_60_) triad dissolved in explicit tetrahydrofuran solvent.^54^ In the meanwhile, the isolated bath model gives rise to significant deviations in nonadiabatic dynamics from the reference, which can be ascribed to the lack of explicit treatment for the correlations between all excited states.^53^ The globally shared bath in the MSH model mimics one or more chemical species on different electronic states immersed in a correlated and heterogeneous environment, and it can also describe the case when some or all electronic states have uncorrelated bath modes, which is represented as orthogonal PES minima, if that is what all-atom simulations dictate. The MSH model’s ability to account for the heterogeneous environment for multiple electronic states, either on the same molecule or belonging to different molecules, makes it a general and consistent Hamiltonian for charge and energy transfer dynamics in complex condensed phases.^55^

Despite the straightforwardness of the MSH model, the collective and delocalized normal modes still lack a clear physical picture to answer which nuclear motion really pushes the reaction forward. A reaction coordinate (RC), if any, provides a clear physical picture of nonadiabatic dynamics, such as an intuitive measure of the reaction progress in terms of the current nuclear distribution and the characteristic time scale of the RC. Searching for the RC of chemical reactions has a long history. Particularly in quantum dynamics, the Caldeira-Leggett (CL) Hamiltonian has been used widely in quantum dissipative systems and superconducting quantum-interference devices (SQUID).^56−58^ Garg, Onuchic, and Ambegaokar applied the CL-type of Hamiltonian with a harmonic reaction coordinate to the two-state electron transfer problem.^59^ Burghardt and co-workers extended the spin-boson model to an effective-mode Hamiltonian, which represents the vibrational modes in terms of a series of effective modes while the rest of the bath modes are truncated as approximate spectral densities in order to reduce the bath dimensionality and computational cost.^60−62^ Recently, Popp et al. reported Frenkel and CT exciton dynamics in oligothiophene aggregates using a reduced-dimensional effective-mode model.^63−65^ Makri proposed a system-bath Hamiltonian in extended dimensions, where the reorganization energies and the PES minima between any pair of sites are identical and independent of site-to-site distance.^66^ However, there is still no clear way to map a heterogeneous and globally shared bath for multiple electronic states such as in MSH model to a well-defined reaction coordinate, which is coupled to a secondary bath.

In this work, we propose a systematic way to transform the MSH model defined in the normal mode basis into the multistate reaction coordinate (MRC) model, where the electronic subspace is coupled only to the RC, and the RC is coupled to the secondary bath modes. The main purpose is to find the MRC Hamiltonian that is exactly equivalent to the MSH Hamiltonian rather than an approximate way. We will test the equivalence between the MRC and the MSH models in two prototypical systems, including the photoinduced CT in the CPC_60_ triad dissolved in explicit solvent^67−71^ and the EET in photosynthetic light-harvesting Fenna–Matthews–Olson (FMO) complex.^40,41,44,45,55,72−76^ We employ several nonadiabatic semiclassical dynamics methods, but our focus here is whether the two models using the same dynamical methods give rise to identical results rather than comparing the accuracy between different dynamical methods. A benefit of RC is that one can easily prepare a nonequilibrium initial nuclear state with predefined shifts with respect to all electronic states.

The remainder of this work is organized as follows. Section 2 presents the construction of the MRC model Hamiltonian by building up from the single-state CL model, the two-state Garg-Onuchic-Ambegaokar (GOA) model, and the MSH model. Section 3 provides simulation details for constructing MRC models for the photoinduced CT in the CPC_60_ triad dissolved in organic solvent as well as EET in photosynthetic light-harvesting FMO complex in *Chlorobaculum tepidum* (*C. tepidum*) and *Prosthecochloris aestuarii* (*P. aestuarii*). Section 4 presents the results and discussion. Section 5 provides the concluding remarks. Appendix A provides the formulas for the equilibrium shifts in the MRC and MSH models.

## 2 Model Hamiltonians

### 2.1 Single-State Caldeira-Leggett Model

The simplest reaction coordinate model is the single-state Caldeira–Leggett (CL) Hamiltonian,^56,57^ which is composed of a primary reaction coordinate, *ŷ*, subject to an arbitrary potential  that can be harmonic or anharmonic, and a collection of secondary harmonic bath modes, , and the primary mode and the secondary bath modes are bilinearly coupled:
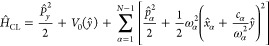
4

### 2.2 Two-State Garg-Onuchic-Ambegaokar Model

The Garg-Onuchic-Ambegaokar (GOA) model was proposed for two electronic states, where the primary/secondary mode representation of the CL Hamiltonian is adopted for each state, and the PES of the primary mode on two states is assumed to be a shifted harmonic potential.^58,59^ The advantage of the GOA model over the spin-boson model is a clear physical picture provided by the reaction coordinate. The GOA Hamiltonian is given by
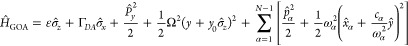
5Here, *ŷ*, , and Ω are the primary reaction coordinate’s mass-weighted coordinate, momentum, and frequency, respectively;  are the mass-weighted coordinates, momenta, and frequencies of the secondary bath modes, respectively; {*c*_α_} is the coupling coefficients between the primary mode and the secondary modes, which should be discerned from the electronic-nuclear couplings  for all normal modes in the spin-boson model; 2*y*_0_ is the distance in equilibrium geometries of the primary mode between the two states, such that the reorganization energy is absorbed entirely to the primary mode, i.e.,
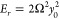
6In the original GOA model, the secondary bath is assumed to follow the Ohmic spectral density given by
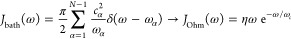
7where η is the friction coefficient and ω_*c*_ is the cutoff frequency. It is worth noting that an asymptotic effective spectral density for the corresponding normal modes in eq 2 was derived for the specific Ohmic bath spectral density at the limit Ω ≪ ω_*c*_ by GOA^59^
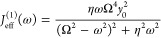
8following the Leggett prescription:^57^
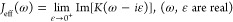
9where with complex *z* and real ω′,
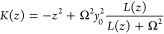
10
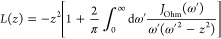
11However, the effective spectral density *J*_eff_^(1)^(ω) in some parameter regions such as Ω ∼ ω_*c*_ could be inaccurate. For example, there can be significant deviation in Fermi’s golden rule rate constant between the spin-boson model with numerically accurate discrete normal modes (eq 2) and the effective spectral density (eq 8),^77^ so caution is advised.

The GOA model and spin-boson model are equivalent and can be transformed from one to the other. To transform from the GOA model to the spin-boson model, one diagonalizes the Hessian matrix of the GOA potential energy surface of either state given by
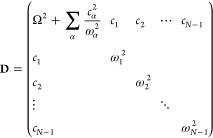
12such that
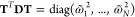
13giving rise to *N* normal-mode frequencies . The spin-boson model can also be expressed in terms of the donor-to-acceptor distance in equilibrium geometry along the normal mode coordinates or {*R*_*j*_^eq^}:^78^
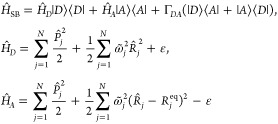
14and the equilibrium geometry distances {*R*_*j*_^eq^} are given by
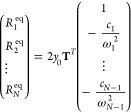
15The reorganization energy is given by
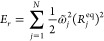
16

Comparing eq 14 with eq 1, we have the relation .

To construct the spin-boson model parameters  for any two states *X*, *Y* (without loosing generality) from all-atom molecular dynamics (MD) simulations, one could use the energy-gap time correlation function (TCF) as an input:
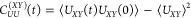
17where *U*_*XY*_ = *V*_*X*_ – *V*_*Y*_ is the energy gap between state *X* and *Y*. The spectral density is related to the energy-gap TCF via^46,47,52,53^
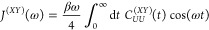
18

The reorganization energy between states *X* and *Y*, *E*_*r*_, scales with the TCF at time 0, :
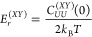
19One can determine the frequencies  and the electronic-nuclear coupling coefficients  or equivalently the equilibrium geometry distances {*R*_*j*_^(*XY*)^} by discretizing the spectral density *J*^(*XY*)^(ω). The discretization into *N* modes is achieved by solving the following equation for :
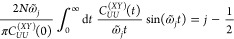
20The corresponding system-bath coupling coefficients, *c*_*j*_, and the equilibrium geometry distances, *R*_*j*_^(*XY*)^, are given by
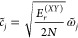
21
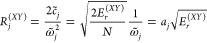
22where
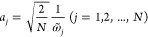
23In this way, each mode contributes to the overall reorganization energy equally and the reorganization energy is given by
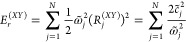
24

### 2.3 Multistate Harmonic Model

Now we consider a general *F*-state Hamiltonian that is given by
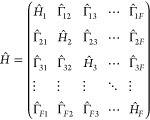
25where the nuclear Hamiltonians of the *F* states in MSH model proposed in ref (53) are given by
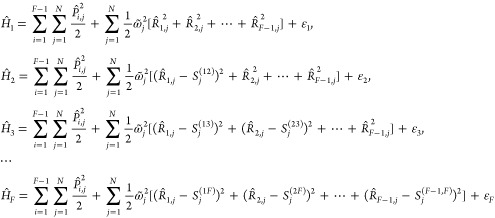
26Here,  are the *N* physical normal-mode frequencies, which can be obtained similarly as the two-state case using eq 20; the index *i* = 1, ..., *F* – 1 labels the *F* – 1 extended subspaces of the physical normal modes resulting in the total nuclear DOF *N*_*n*_ = (*F* – 1) × *N*; {*S*_*j*_^(*XY*)^|1 ≤ *X* < *Y* ≤ *F*} are the equilibrium (horizontal) shift components for the PES of state *Y* along the *j*-th mode; {ε_*X*_|X = 1, ..., *F*} are the energy minima (vertical shifts) of the PESs of the different electronic states;  are the electronic couplings and  is constant under the Condon approximation.

As detailed in ref (53), we introduced the general way for constructing the MSH model Hamiltonian from multistate all-atom MD simulations and quantum chemistry calculations. In short, one needs excited-state electronic structure calculations to determine the excitation energy and the charge distribution for each electronic state, and MD simulations to determine *F*(*F* – 1)/2 energy-gap TCFs between all possible pairs of states, namely {*C*_*UU*_^(*XY*)^(*t*)|*X* < *Y*}. From these energy-gap TCFs, we can compute all of the reorganization energies using eq 19.

As an important feature of the MSH model, the dimensionality of each normal mode is extended from 1 to *F* – 1 such that the equilibrium positions of all *F* electronic states form a polyhedron with *F* vertices, and the equilibrium distances between any pair of states satisfy the reorganization energy restrictions in eq 22. In particular, the *F*(*F* – 1)/2 sets of equilibrium shifts
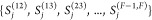
27need to satisfy the *F*(*F* – 1)/2 reorganization energy restrictions that require the equilibrium geometry distances (the polyhedron edges) between each pair of states proportional to
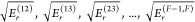
28In this way, we can achieve a consistent account for all of the reorganization energies between *F*(*F* – 1) pairs of states obtained from all-atom simulations. Without extending the spatial dimensionality, the *F*(*F* – 1)/2 reorganization energy restrictions cannot be satisfied simultaneously. The MSH equilibrium shifts {*S*_*j*_^(*XY*)^} are given in Appendix A.

Generally, setting {*S*_*j*_^(*i*,*X*)^} ≡ 0 when *i* ≥ *X*, we can rewrite the nuclear Hamiltonian of the electronic state *X* in eq 26 as
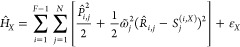
29

The electronic couplings {Γ_*XY*_} can be obtained from the fragment charge difference (FCD) method.^79^ The energy minima {ε_*X*_} are determined from their difference, or the reaction free energies Δ*E*^(*XY*)^, which can be estimated with the reorganization energy and the energy-gap average from equilibrium MD simulations on the PES of the initial electronic state^53^
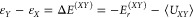
30

### 2.4 Multistate Reaction Coordinate Model

In this section, we propose the multistate reaction coordinate (MRC) model and show that the MSH model can be translated to the equivalent MRC model. We expect the MRC model to have the same DOF as the MSH model, which is defined in *F* – 1 extended dimensions for each normal mode in an *F*-state system. First, starting from the *F*-state Hamiltonian in eq 25, we propose the CL or GOA type of primary/secondary potential for each nuclear subspace in each electronic state , where *i* = 1, ..., *F* – 1, and the nuclear Hamiltonian of the *X*-th electronic state is given by
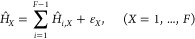
31where the *i*-th subspace component is (*S*^(*i*,*X*)^ ≡ 0 when *i* ≥ *X*)
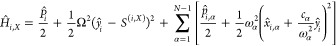
32

Consider the first two states in the first subspace of the MSH model, which is reduced to the asymmetric spin-boson model in eq 14 and the potential energy part is
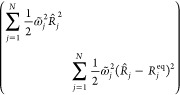
33We apply the Householder reflection^80^ procedure proposed by Wang and Thoss to transform the normal-mode picture into the primary/secondary-mode picture.^27^ The reaction coordinate *ŷ* with normalization factor κ is assigned to be the linear term  in eq 14 that differs from two states such that
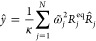
34
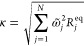
35By definition, the reorganization energy between these two states is the energy difference between the acceptor-state potential energies evaluated at the equilibrium position of the donor surface and the minimum of the acceptor potential energy. For the two-state primary/secondary-mode case below
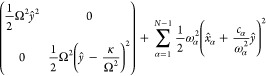
36we expect the reorganization energy to be equal to eq 16, namely
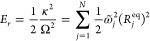
37which leads to primary mode frequency
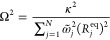
38

Using eqs 36 and 37, we have the equilibrium position distance of the primary mode between the two states
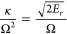
39It is noted that the primary mode *ŷ* and its characteristic frequency Ω do not scale with *R*_*j*_^eq^, which will be used in the multistate extension. Denoting the expansion coefficients of the primary mode as , we express the reaction coordinate vector in the original normal mode basis {|*R*_1_⟩, |*R*_2_⟩, ..., |*R*_*N*_⟩} as
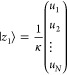
40The Householder reflection operator is defined as
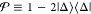
41where
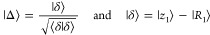
42The  reflects any vector about the plane perpendicular to the difference between the original normal mode |*R*_1_⟩ and the reaction coordinate |*z*_1_⟩, and it satisfies . Applying this reflector to all the normal mode vectors, we have a new set of orthonormal vectors:
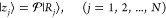
43Denoting the coordinates of the normal modes and the new basis as column vectors  and , respectively, we express the harmonic potential about its equilibrium position as
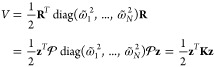
44Here, the new Hessian **K** in the {|*z*_*j*_⟩} basis is defined as
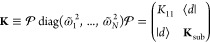
45where **K**_sub_ is the last (*N* – 1) × (*N* – 1) block of Hessian matrix **K**, (*N* – 1)-dim vector ⟨*d*| = (*K*_21_, *K*_31_, ..., *K*_*N*1_), and element
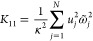
46To get orthogonal secondary bath modes, we diagonalize the sub-Hessian matrix **K**_sub_ by orthogonal transformation and obtain the bath frequencies
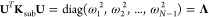
47which leads to the block transformation
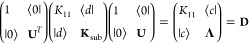
48Here, the primary/secondary coupling coefficients are
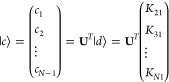
49the primary mode , and the secondary modes (*x*_1_, ..., *x*_*N*–1_) are transformed as
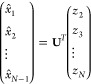
50and the potential in eq 44 is finally transformed to the primary/secondary-mode form as below
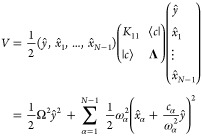
51

Next, we consider the *F*-state case. Since *ŷ*, Ω, and **K** do not scale with *R*_*j*_^eq^, the primary/secondary couplings {*c*_α_} do not change with the reorganization energy, so the reorganization energy for different pairs of states will be absorbed to the equilibrium positions of the primary mode. In the MSH model, the equilibrium shifts of the normal modes are built to scale with square roots of the reorganization energies, so we can apply the same polyhedron geometry to the primary mode or the reaction coordinate in the MRC model in *F* – 1 dimensions. The transformation matrix **U** can be chosen to be the same in different subspaces *i* = 1, ..., *F* – 1, since flipping the sign of any eigenvector does not affect the diagonalization or the eigenvalue. Using eq 39, we have the equilibrium shifts for the primary mode as below (see eq 65 for more details).
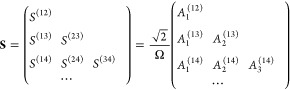
52Now, we have obtained the model parameters including Ω, {ω_α_}, {*c*_α_}, {*S*^(*XY*)^} that define the MRC model and rewrite eqs 31 and 32 as below
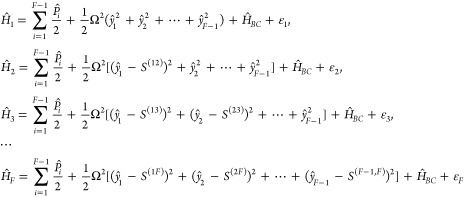
53where  for the secondary bath and the primary/secondary coupling is given by
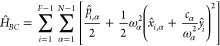
54

We could translate between the MSH model and the MRC model through Hessian matrix diagonalization, where the Hessian matrix **D** is the same as eq 12 for each subspace , (*i* = 1, ..., *F* – 1). The instantaneous positions (*R*_*i*,1_, *R*_*i*,2_, ..., *R*_*i*,*N*_) of the MSH model can be translated into or from the corresponding MRC configuration (*y*_*i*_, *x*_*i*,1_, ..., *x*_*i*,*N*–1_) using
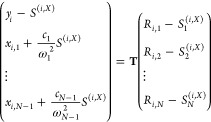
55

Here, the transformation matrix **T** is given in eq 13, which requires the knowledge of primary frequency Ω, secondary bath modes’ frequencies {ω_α_} and their couplings with the primary mode {*c*_α_}.

It is important to note that transforming from a primary/secondary coupled MRC model to an uncoupled normal-mode MSH model is straightforward, which can be obtained by diagonalizing the Hessian **D** as shown in eq 13. However, there are many ways to transform from normal mode parameters in the MSH model to a primary/secondary coupled MRC model, and the current approach described above using Householder reflection provides an effective way of achieving this goal. Moreover, knowing the explicit form of how the primary modes  couple with the secondary bath  and  does not depend on electronic states is important for verifying that  indeed serves as RC. Also, it is equally important to prove the equivalence and transformations between MSH and MRC models analytically and numerically, and we will show the numerical comparison in section 4. In principle, this primary/secondary partitioning procedure is not limited to the first layer of RC, so one could keep applying this procedure to achieve a hierarchy of RC layers. The MRC model Hamiltonian provides a convenient and intuitive way of understanding nonadiabatic dynamics in multistate systems.

## 3 Simulation Details

### 3.1 Model Preparation

**Figure 2:**
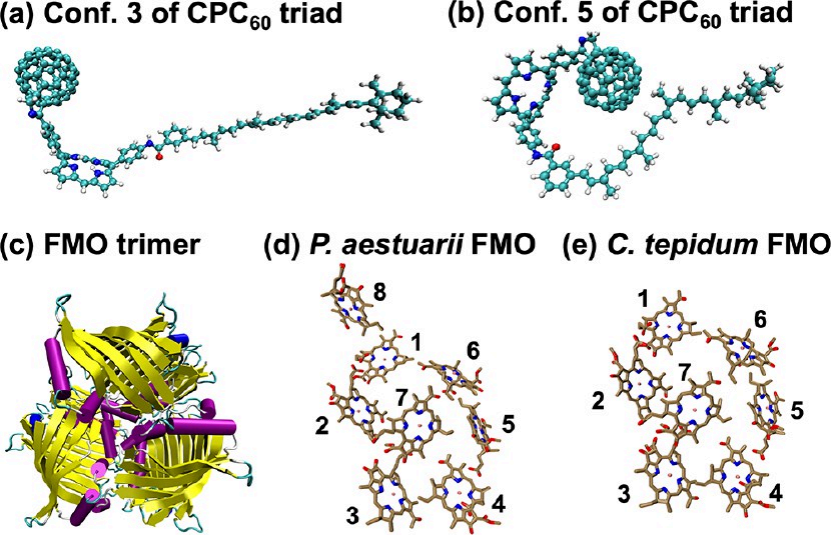
Molecular conformations of carotenoid-porphyrin-C_60_ (CPC_60_) triad including conformation #3 (a) and conformation #5 (b) as well as the light-harvesting Fenna-Matthews-Olson (FMO) complex trimer of *P. aestuarii* (c), 8 BChl sites in *P. aestuarii* FMO complex, and 7 BChl sites in *C. tepidum* FMO complex.

We construct the MRC and the MSH models for photoinduced charge transfer dynamics in two CPC_60_ triad conformations with four electronic states, {ππ*, CT1, CT2, G}, where conf. #3 was previously reported in ref (54). and conf. #5 is a new one (see Figure 2). Here, in the donor-bridge-acceptor type of organic photovoltaic CPC_60_ triad, the ππ* state is the porphyrin-localized excited bright state, CP*C_60_, CT1 state is a partially charge-separated state, CP^+^C_60_^–^, CT2 state is a fully charge-separated state, C^+^PC_60_^–^, and G is the ground electronic state. Confirmations #3 and #5 were sampled from molecular dynamics (MD) simulations on the ground state and selected since their CT2 state gets populated in the first few picoseconds after the initial photoexcitation. CT dynamics is conformation-specific, so one might have to resort to a statistical description for an ensemble of conformations at finite temperatures, such as the CT landscape predicted by machine learning.^81^ In both MRC and MSH models, the number of physical normal modes *N* = 200 and the total nuclear DOF is *N*_*n*_ = (*F* – 1) × *N* = 600. The energy minima, interstate couplings, energy corrections, and reorganization energies are summarized in Table 1. The mode frequencies and the equilibrium shifts along with other MRC model parameters are provided in the Supporting Information.

**Table 1:** Energy Minima, Interstate Couplings, Energy Corrections, and Reorganization Energies for Confirmations 3 and 5^a^

	conf. 3	conf. 5
ε_1_(ππ*)	0	0
ε_2_(CT1)	–0.828	–0.758
ε_3_(CT2)	–0.640	–1.128
ε_4_(G)	0	0
*W*_1_(ππ*)	0.728	1.551
*W*_2_(CT1)	–2.103	–0.697
*W*_3_(CT2)	–2.131	–0.650
*W*_4_(G)	0	0
Γ_12_	–1.5 × 10^–2^	8.1 × 10^–2^
Γ_13_	7.2 × 10^–3^	4.1 × 10^–3^
Γ_23_	–2.9 × 10^–2^	–3.2 × 10^–3^
Γ_*Y*4_	0	0
*E*_*r*_^(12)^	7.880	6.464
*E*_*r*_^(13)^	11.39	18.68
*E*_*r*_^(14)^	0.9202	20.22
*E*_*r*_^(23)^	3.546	0.3096
*E*_*r*_^(24)^	21.23	7.920
*E*_*r*_^(34)^	26.42	18.95
Ω	198.93	228.47

a Energy minima ε_*X*_ and energy corrections *W*_*X*_ of four electronic states, electronic couplings Γ_*XY*_ and reorganization energies *E*_*r*_^(*XY*)^ (unit kcal/mol) between different pairs of states in the two conformations (#3 and #5) of the triad (unit in eV), as well as the reaction coordinate frequencies Ω (unit in cm^–1^). Here, electronic states *X* = 1, 2, 3, and 4 correspond to ππ*, CT1, CT2, and ground (G) states, respectively. The electronic couplings between any excited states (*Y* < 4) and the ground state are assumed to be zero.

To obtain the MRC model parameters, we performed MD simulations for calculating the energy-gap time correlation functions (eq 17), and thus the spectral densities (eq 18) and the reorganization energies (eq 19). MD simulation of a 70 Å × 70 Å × 70 Å periodic box containing a triad on the ππ* state and 2700 THF solvents at 300 K was done using the PMEMD program of AMBER20 package.^82^ Particle Mesh Ewald summation was used to calculate the electrostatic interactions.^83^ The van der Waals and real-space PME cutoff radii were set to 9.0 Å. The time step of the MD simulation was set to *δt* = 1 fs. The triad conformation was kept rigid with a steep harmonic constraint of force constant 100 kcal mol^–1^ Å^–2^, and SHAKE^84^ algorithm was used to constrain all covalent bonds involving hydrogen atoms. The MD simulation follows the steps below. First, the system was minimized for 1000 steps with the steepest descent method, which is followed by 39000 steps of conjugate gradient minimization with an initial step length of 0.01 ps. Second, the system was heated from 0 to 300 K for 20 ps and relaxed for 80 ps at 300 K under the Langevin thermostat with a collision frequency of 2.0 ps^–1^. Third, the system was equilibrated under isothermal–isobaric (NPT) ensemble at 300 K and 1 bar for 500 ps with Langevin thermostat with a collision frequency of 1.0 ps^–1^ and Berendsen barostat with a pressure relaxation time of 0.5 ps and compressibility of 4.46 × 10^–5^ bar, and the final averaged box size is 71.524 Å × 71.595 Å × 71.500 Å. Fourth, 200 independent initial conditions including configurations and velocities were sampled from a canonical (NVT) ensemble at 300 K with a Langevin thermostat with a collision frequency of 1.0 ps^–1^ every 20 ps after re-equilibration of 500 ps. Fifth, starting from each initial condition, after relaxation of 50 ps, a microcanonical (NVE) trajectory of 100 ps was generated and configurations were saved every 5 fs, which was used to recalculate the potential energies on other electronic states using
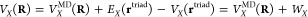
56

**Figure 3:**
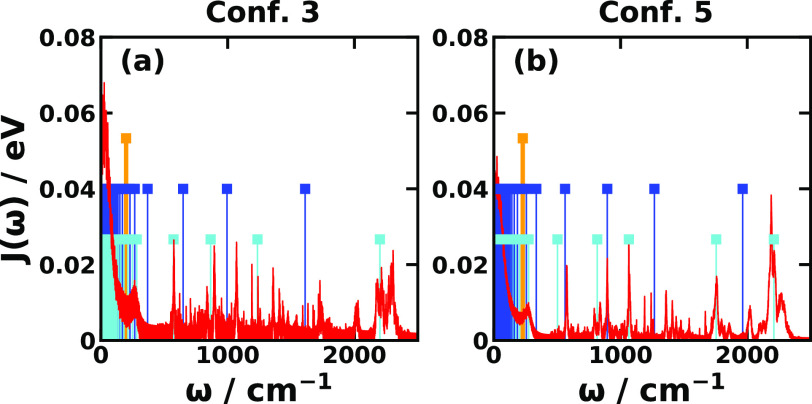
Spectral density (red), reaction coordinate frequency (orange), and secondary bath mode frequencies (blue) of MRC model, and normal-mode frequencies of MSH model (cyan) for CPC_60_ triad conformations #3 and #5 obtained with all-atom simulations at 300 K.

Here, *V*_*X*_^MD^(**R**) is the MD potential energy of the whole system with the triad on its *X* excited state calculated by the force field in the general AMBER force field (GAFF)^85^ form but atomic charges replaced with those from electronic structure calculation. *E*_*X*_(**r**^triad^) is the energy of the gas-phase triad on the *X* excited state obtained from electronic structure calculation, *V*_*X*_(**r**^triad^) is the gas-phase triad energy on the *X* excited state obtained from the force field, and *W*_*X*_ is the energy correction for the excitation energy for the triad on the *X* excited state.^71^ Sixth, using the 200 independent NVE trajectories, we calculated the energy gap TCFs *C*_*UU*_^(*XY*)^(*t*) with a time interval of 5 fs and up to 11 ps, from which we obtain the spectral densities (see Figure 3) and reorganization energies (see Table 1) between pairs of states. The resulting reaction coordinate frequencies Ω for conf. #3 and conf. #5 are given in Table 1 and they range from 200 to 228 cm^–1^ corresponding to typical intermolecular motion in the liquid phase. The equilibrium shift matrix (in a.u.) defined in eq 52 for conf. #3 and conf. #5 are given respectively by
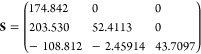
57
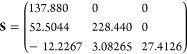
58

The electronic couplings between different states {Γ_*XY*_} (see Table 1) were obtained by using the FCD method. The energy minima {ε_*X*_} (see Table 1) were obtained from time-dependent density functional theory (TDDFT) calculations^71,86^ and the average energy gap from MD simulation using eq 30. The TDDFT calculation for the triad was performed with the range-separated hybrid (RSH) Baer-Neuhauser-Livshits (BNL) functional (range-separation parameter ω = 0.157) in SV basis using QChem 4.4.^87^ Since the ground state is only for initial nuclear sampling and it does not have electronic couplings with the three excited states in the model, the energy minimum parameter ε_*G*_ does not affect the dynamics and thus can be chosen arbitrarily.

The MRC models for excitation energy transfer in the photosynthetic light-harvesting FMO complex were constructed for 7 bacteriochlorophyll (BChl) sites in the *C. tepidum* FMO complex and 8 BChl sites in the *P. aestuarii* FMO complex (see Figure 2), corresponding to Protein Data Bank (PDB) identification numbers 3ENI and 3EOJ, respectively. The FMO complexes in *C. tepidum* and *P. aestuarii* have different absorption spectra and thus different excitation energies and EET dynamics.^40,41,44,45,55,72−76^ In fact, the *C. tepidum* FMO complex also has the eighth BChl site, but since historically the 7-site model has been widely used for quantum dynamics calculations, we decided to test the 7-site model.^40^ The MSH model parameters for 7-site *C. tepidum* and 8-site *P. aestuarii* FMO complexes were reported in ref (55). The model is based on all-atom quantum-mechanics/molecular-mechanics (QM/MM) simulation of the entire FMO trimer in explicit water.^44,45^ We refer the reader to ref (55) for more model parameters including the site energies, electronic couplings, and pairwise reorganization energies. It is noted that with the additional ground state the 7-site and 8-site MSH/MRC models correspond to *F* = 8 and *F* = 9, respectively. The physical nuclear modes for both *C. tepidum* and *P. aestuarii* FMO complexes are *N* = 100, and total nuclear DOF *N*_*n*_ = (*F* – 1) × *N*. Following the procedure introduced in the previous section, we translated the MSH model to the MRC model and obtained that the reaction coordinate frequencies for the 7-site *C. tepidum* and the 8-site *P. aestuarii* FMO complexes are 1187.2 and 507.68 cm^–1^, respectively. The MRC model parameters for both FMO complexes are provided in the Supporting Information.

### 3.2 Nonadiabatic Dynamics Simulations

In most photoinduced chemical dynamics, the initial state of the overall system can be assumed to be given by the overall density operator
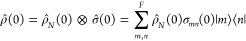
59where the initial nuclear and electronic reduced density matrices (RDM) are given by  and , respectively, and Tr_*e*_[·] and Tr_*N*_[·] denote partial trace over the electronic and nuclear Hilbert spaces, respectively. The dynamics of the electronic RDM at time *t* after the photoexcitation is of interest, i.e.,
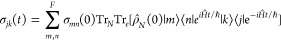
60where the diagonal elements σ_*jj*_(*t*) are electronic populations and the off-diagonal elements σ_*jk*_(*t*) (*j* ≠ *k*) are electronic coherences.

Usually, in photoinduced CT or EET processes, one usually considers the case of starting from a population of a certain electronic state, say, σ̂(0) = |m⟩⟨m| or σ_*mm*_(0) = 1 and all other initial RDM elements vanish. In our simulations, we start from the |ππ*⟩⟨ππ*| population for the triad that mimics the result of vertical excitation from the ground state (or other nonequilibrium state) to the ππ* state, and the FMO complex in the initial excitation populates the BChl site 1 in both *C. tepidum* and *P. aestuarii* FMO complexes, namely |1⟩⟨1|.

The initial nuclear conditions including positions and momenta correspond to the Wigner transform of , which can be the ground state or an arbitrary state:
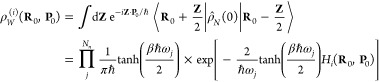
61where β = 1/*k*_B_*T* and *T* = 300 K, *N*_*n*_ is the total nuclear DOF, and *H*_*i*_(**R**_0_, **P**_0_) is the classical harmonic nuclear Hamiltonian that dictates the initial nuclear state. To have consistent initial nuclear conditions, we sample in the MSH model and transform to the MRC model as shown in eq 55.

The nonadiabatic dynamics of the MSH and the MRC models for triad and FMO systems were simulated using nonadiabatic semiclassical dynamics methods that were recently employed,^53,54^ including Ehrenfest mean-field (MF),^88^ resolution of identity linearized semiclassical mapping No. 1 (RI-LSC1),^38,39^ symmetrical quasi-classical dynamics with triangle window (SQC),^35^ and spin-mapping approach in W scheme (SPM).^89^ These approaches could be regarded as mapping dynamics methods where the electronic DOF are represented as the Meyer-Miller-Stock-Thoss mapping variables,^90,91^ if we consider MF as the special case when the zero-point energy parameter vanishes. It should be noted that the main point here is to test the equivalence between MSH and MRC models under different methods rather than discussing the performance or accuracy of different dynamical methods. In our nonadiabatic simulations, the nuclear DOF is propagated using the velocity-Verlet algorithm, electronic mapping variables are propagated using the fourth-order Runge–Kutta algorithm, and mapping variables update 20 times per nuclear step. For both MSH and MRC models of the triad system, the nuclear time step was chosen as *δt* = 0.1 fs and the electronic population dynamics was obtained by averaging over 10^5^ trajectories, except for the RI-LSC1 method, which was over 10^6^ trajectories; for both MSH and MRC models of the FMO complex, the nuclear time step was chosen as *δt* = 1 fs and the electronic population dynamics was obtained by averaging over 10^6^ trajectories. The computational costs for both MSH and MRC models are almost the same; for example, for a triad conformation 140 core-hours (e.g., Intel Xeon Gold 6132 Central Processing Unit with 2.6 GHz base frequency) would be required to sample over 10^6^ trajectories of 10 ps each.

## 4 Results and Discussion

In this section, we first present the photoinduced CT dynamics in the CPC_60_ triad of two different conformations in the solution phase as well as the EET dynamics in photosynthetic light-harvesting FMO complexes in *C. tepidum* and *P. aestuarii* obtained with corresponding MSH and MRC models at 300 K. It is noted that the main focus here is to test the equivalence between MSH and MRC models, which is expected since the mathematical transformation between the two models is exact.

**Figure 4:**
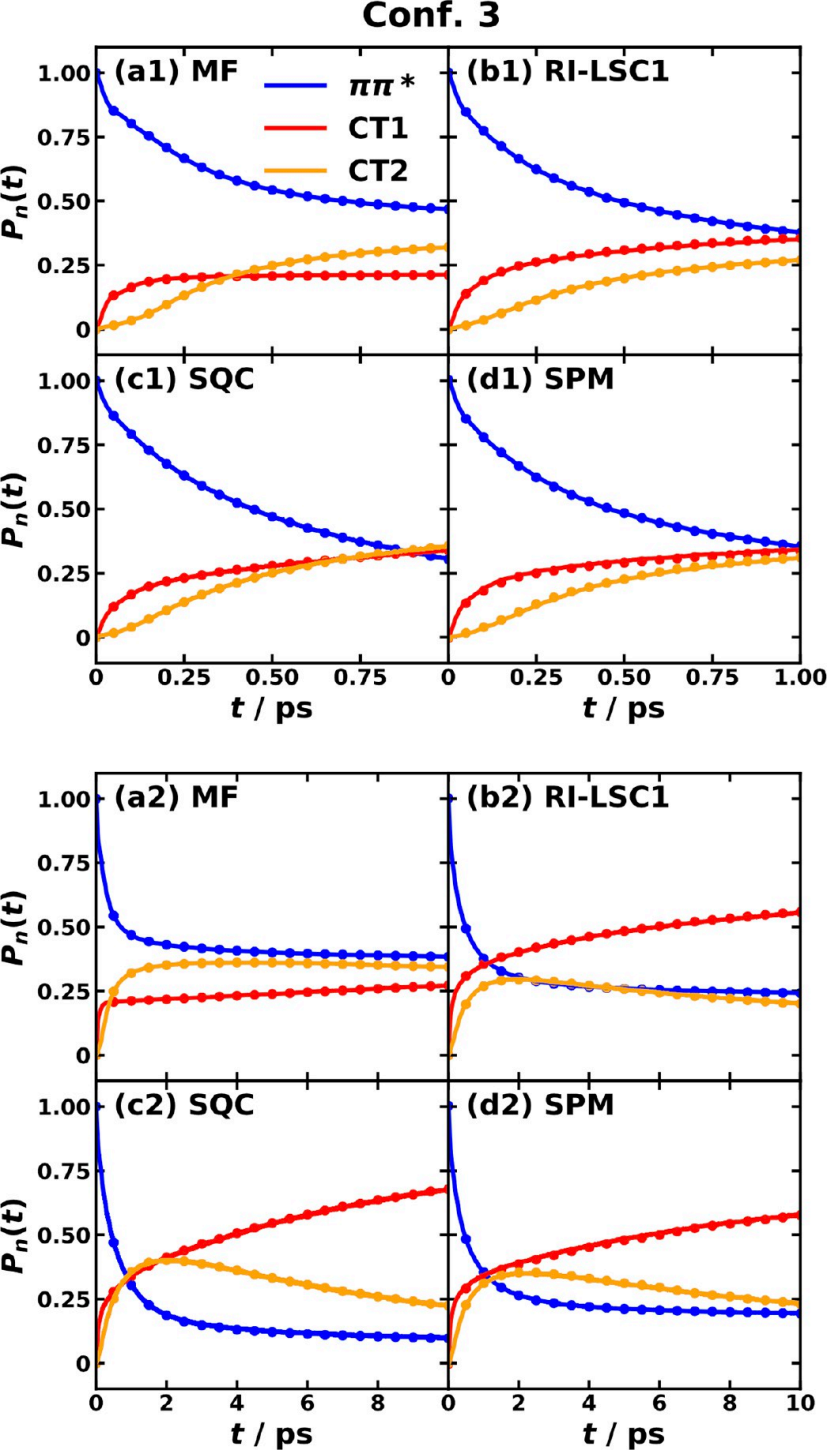
Photoinduced CT population dynamics for MSH model (solid lines) and MRC model (dots) of the CPC_60_ triad conformation #3 obtained with different semiclassical dynamics at 300 K. The initial electronic state is |ππ*⟩⟨ππ*| and the initial nuclear state is in thermal equilibrium with the ground state potential energy surface.

**Figure 5:**
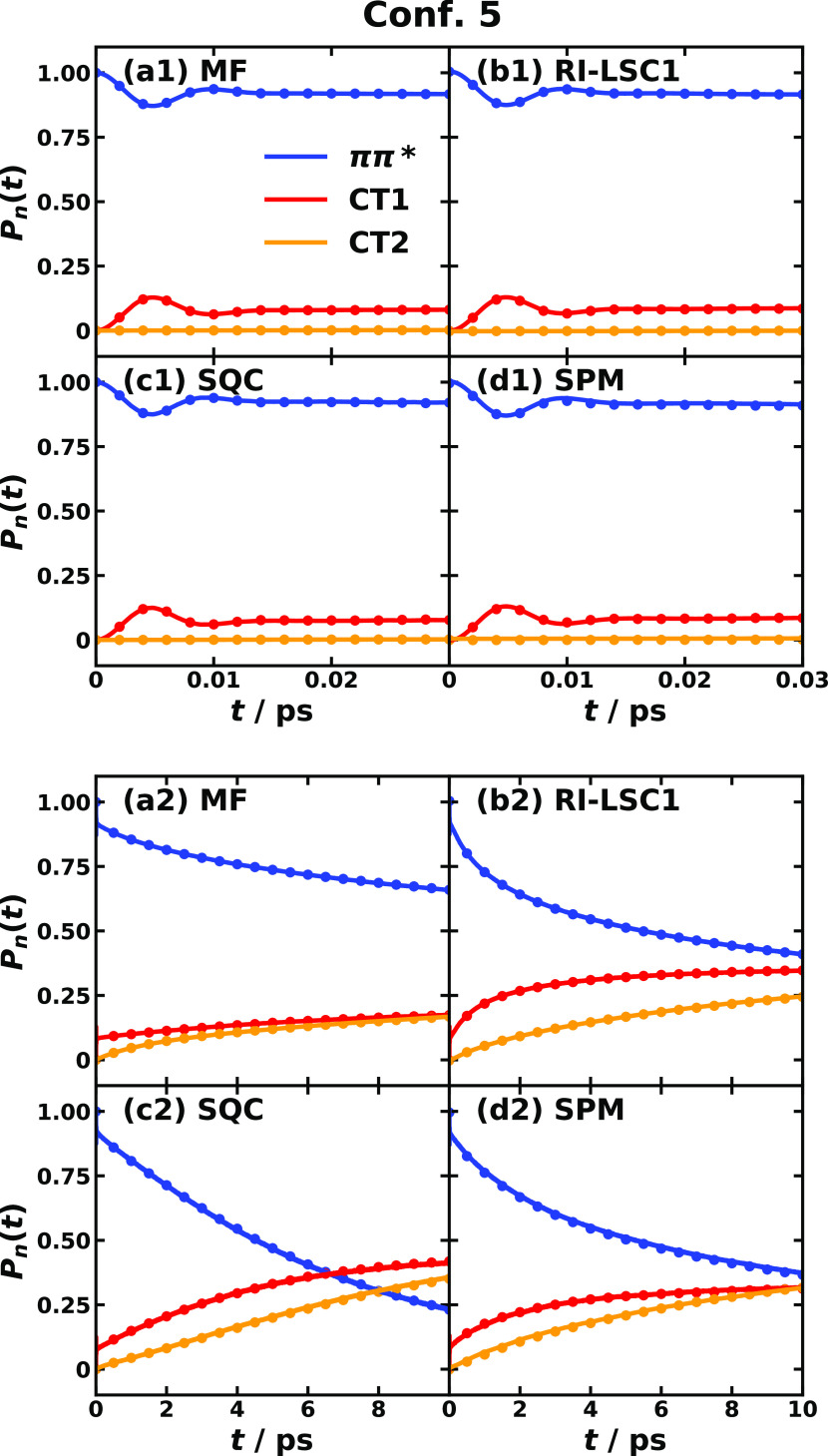
Photoinduced CT population dynamics for the MSH model (solid lines) and MRC model (dots) of the CPC_60_ triad conformation #5 obtained with different semiclassical dynamics at 300 K. The initial electronic state is |ππ*⟩⟨ππ*| and the initial nuclear state is in thermal equilibrium with the ground state potential energy surface.

Figures 4 and 5 display the comparison between MSH and MRC models for conf. #3 and conf. #5 of the CPC_60_ triad, respectively. It is evident that the nonadiabatic dynamics produced by using MSH and MRC models are identical within the same dynamical method, which validates the equivalence between the MSH and MRC model Hamiltonians. As we checked, the numerical results of the electronic populations obtained with MSH and MRC Hamiltonians are the same within 11 significant figures in the first tens of steps and within 7 significant figures at 1 ps. It is believed that this small difference in populations between MSH and MRC models is due to the numerical error in the computer’s double precision, rather than the transformation formula.

For triad conf. #3, the short- and long-time population dynamics are plotted as the upper and lower panel groups of Figure 4. What we observe is that after the photoexcitation putting the system on the ππ* excited state but the nuclear DOF are still in equilibrium with the ground state PES, the electronic state population on ππ* state starts to decay to the other two CT states. In the first 1 ps, the populations of CT1 and CT2 grow to a similar level of about 0.3 crossing with the initial ππ*, while the response time of CT1 is quicker than that of CT2. Then in the longer time scale of 10 ps, the population of CT1 continues to increase gradually, whereas the population of CT2 starts to decrease slightly in RI-LSC1, SQC, and SPM dynamics but not in MF dynamics, which is believed to be less accurate than the other methods. The nonadiabatic dynamics predicted by the MSH model has been demonstrated to agree with that obtained with the explicitly all-atom multistate Hamiltonian in ref (54). The agreement among MSH, MRC, and all-atom Hamiltonians in combination with the same dynamical method suggests the effectiveness of the simplified MSH and MRC models in studying the nonadiabatic dynamics in complex condensed-phase systems.

For triad conf. #5, two separated time scales are observed as shown in Figure 5. Here, an ultrafast population oscillation between the initial ππ* state and CT1 state in the first 20 fs after photoexcitation is shown in the upper panels of Figure 5, followed by a slow population transfer from ππ* to CT1 and CT2 in the 10 ps scale shown in the lower panels of Figure 5. For ultrafast dynamics, all four methods give rise to the same result, while for longer-time dynamics, MF predicts a slower transfer rate compared with the other three methods. Besides the electronic RDM dynamics including populations and coherences, we also confirm that the nuclear motions are identical by using MSH and MRC models under the same dynamical method, which suggests that the MSH and MRC models are fully equivalent.

**Figure 6:**
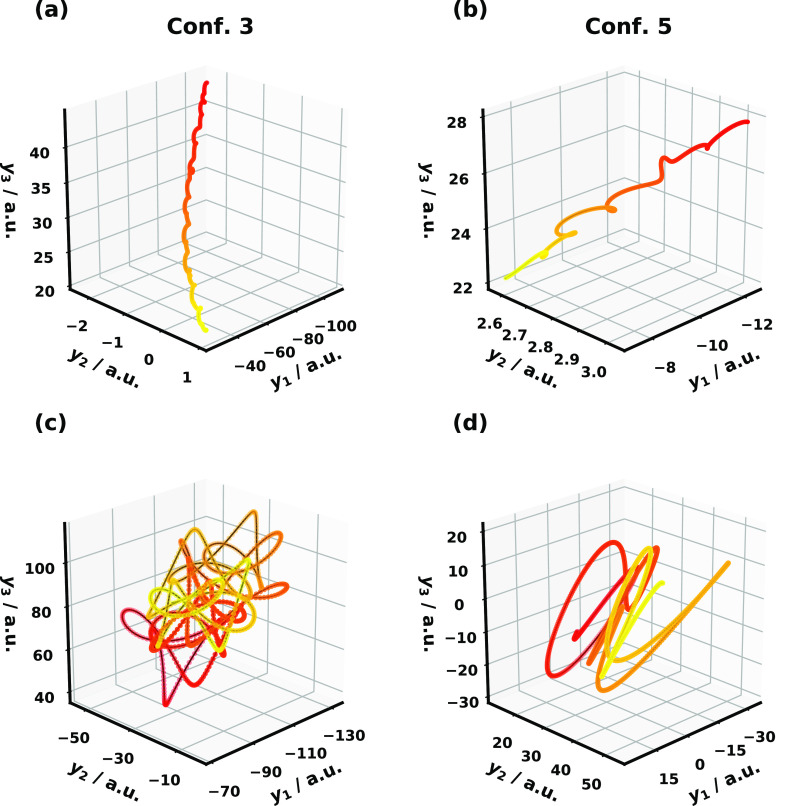
Reaction coordinate trajectory for MRC models of CPC_60_ triad conformation #3 (left) and conformation #5 (right) obtained with the SQC method. Here, top panels (a) and (b) are averaged over 10^5^ trajectories; bottom panels (c) and (d) are single sampled trajectories; left panels (a) and (c) show the first 0.3 ps in conformation #3; right panels (b) and (d) show the first 0.1 ps of conformation #5; the color gradient from red to yellow corresponds to the initial and to the final times.

A benefit of MRC models is that the trajectory of the RC is readily available. Figure 6 shows the averaged and single sampled RC trajectories for MRC models of triad conf. #3 and conf. #5 in the first 0.3 and 0.1 ps after the photoexcitation, respectively. The RC trajectories offer an intuitive picture for understanding the photoinduced CT dynamics. For example, the highly averaged RC trajectory exhibits a smooth pathway with a clear trend determined by the inherent driving force, whereas a single trajectory displays a wildly oscillatory feature that masks the trend. Fortunately for all four-state MRC models, we could render the reaction coordinate in a 3-dimensional plot; for any system with more than four states, it would be difficult to plot all RC components together, so one would have to consider the components separately.

**Figure 7:**
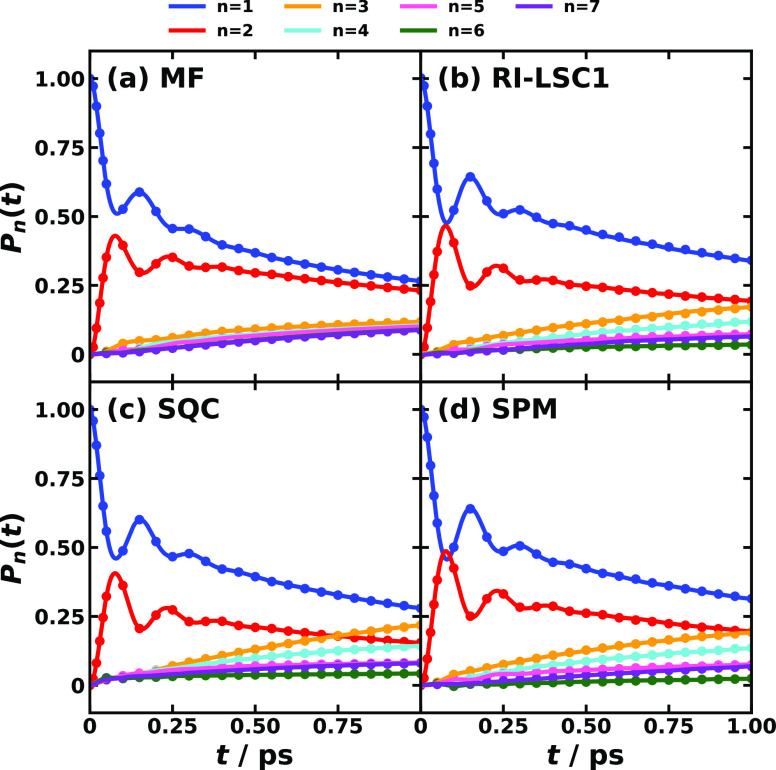
Photoinduced EET population dynamics for MSH model (solid lines) and MRC model (dots) of the 7-site *C. tepidum* FMO complex obtained with different semiclassical dynamics at 300 K. The initial electronic state is |1⟩⟨1| and the initial nuclear state is in thermal equilibrium with the ground state potential energy surface.

**Figure 8:**
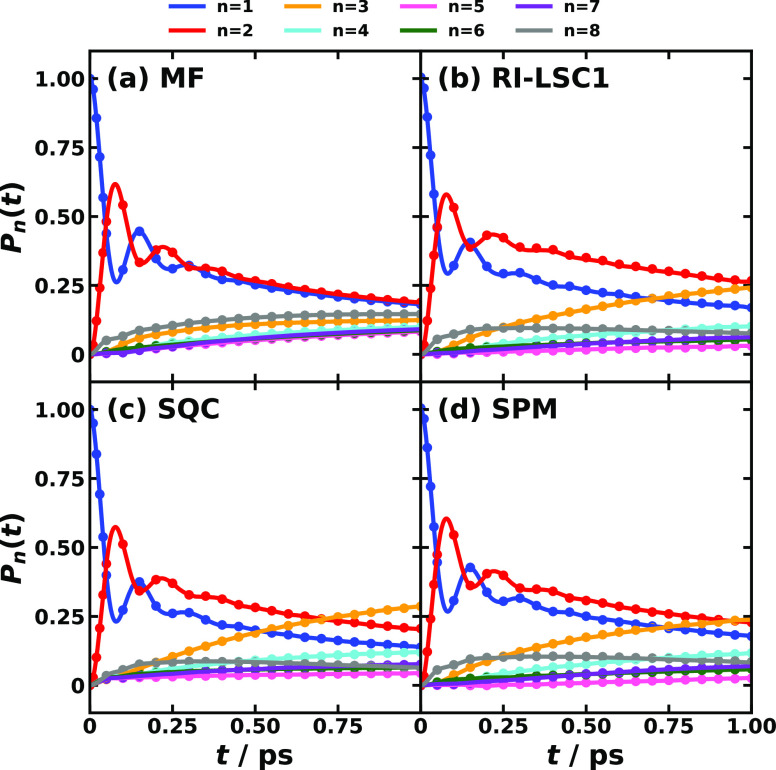
Photoinduced EET population dynamics for MSH model (solid lines) and MRC model (dots) of the 8-site *P. aestuarii* FMO complex obtained with different semiclassical dynamics at 300 K. The initial electronic state is |1⟩⟨1|, and the initial nuclear state is in thermal equilibrium with the ground state potential energy surface.

We now turn our attention to EET dynamics in photosynthetic FMO complexes in *C. tepidum* and *P. aestuarii* as shown in Figures 7 and 8, respectively. First of all, EET dynamics using the MRC model coincide with that using the MSH model^55^ in all cases considered here, including in both *C. tepidum* and *P. aestuarii* FMO complexes using different nonadiabatic dynamics methods. Second, we do observe different EET pathways in *C. tepidum* and *P. aestuarii* FMO complexes even if the initial excitation is localized on BChl site 1, where the populations of BChl site 1 and BChl site 2 do not cross in *C. tepidum* FMO but they do so in *P. aestuarii* FMO. Third, the eighth BChl site is involved in the EET pathway, as shown in the *P. aestuarii* dynamics where the BChl site 8 gets populated in the first 0.2 ps after initial photoexcitation, especially considering that the spatial location of the BChl site 8 is further away from the final populated BChl site 3. Fourth, both MSH and MRC models incorporate the treatment of static disorder in state-pairwise reorganization energies, realistic spectral density, and state-dependent system-bath couplings. Our previous calculations exhibit that the MSH model with the heterogeneous protein environment description gives rise to a bit slower EET dynamics than the conventional Frenkel exciton model.^55^

We briefly discuss the differences between the MSH/MRC model and the isolated bath model, such as the Frenkel exciton model. The conventional Frenkel exciton model has an overall Hamiltonian given by eq 25 (the same as MSH/MRC model), but the nuclear Hamiltonian of the *X*-th electronic state is given by
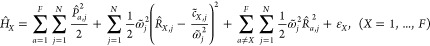
62and the ground-state bath Hamiltonian used for determining the initial nuclear condition is given by
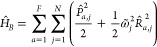
63Here, the system–bath coupling coefficients  could in principle depend on the site *a*, but they are determined only by the spectral density and reorganization energy between the excited state on the *a*-th site and its ground state, i.e., black double arrows for *E*_*r*_^(*Xg*)^ in Figure 1(a). The resulting Frenkel exciton model thus has *F* independent baths that are coupled to each chromophore site, resulting in a total nuclear DOF of *F* × *N*. Thus, it is impossible to incorporate the relations between two excited states in the isolated bath models, such as the Frenkel exciton model. The MSH/MRC models, on the other hand, incorporate all the possible *F*(*F* – 1)/2 state-pairwise reorganization energies, i.e., orange arrows for *E*_*r*_^(*XY*)^ and black arrows for *E*_*r*_^(*Xg*)^ forming polyhedron in *F* – 1 dimensions in Figure 1(b).

**Figure 9:**
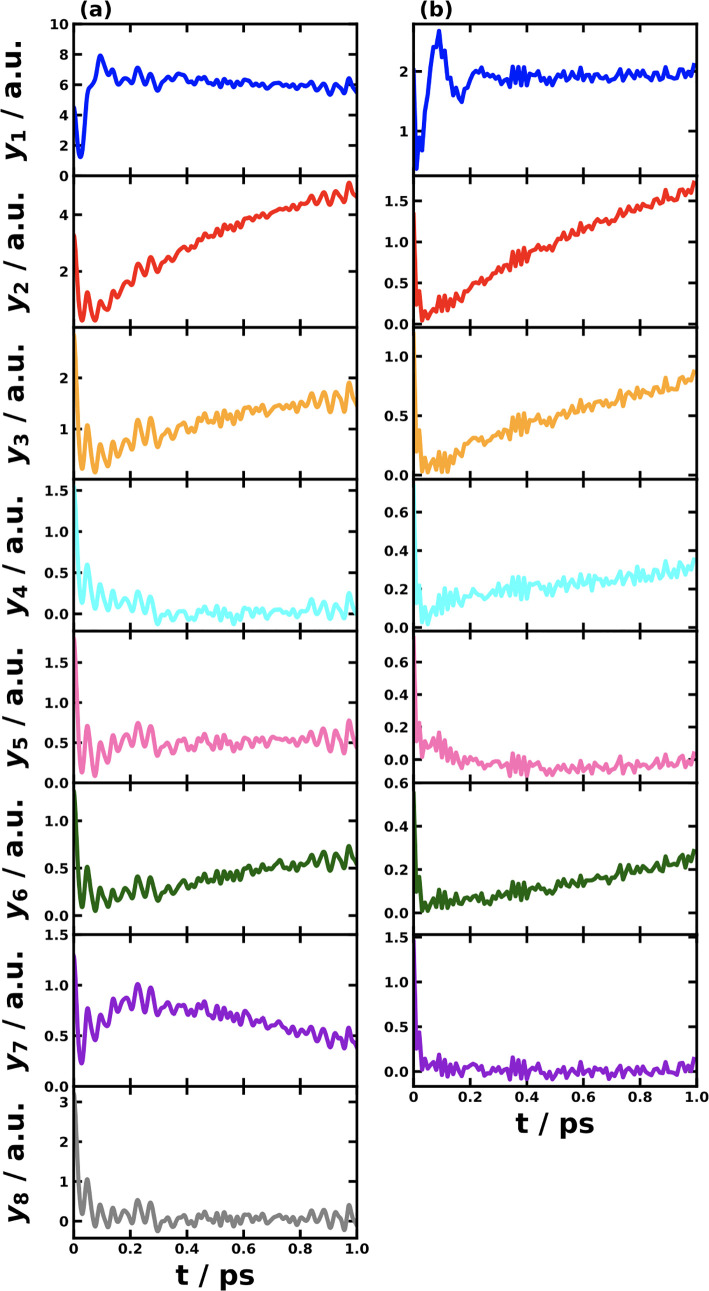
Reaction coordinate trajectories for MRC models of the 8-site *P. aestuarii* FMO complex (a) and 7-site *C. tepidum* FMO complex (b) obtained with averaging 10^6^ SQC trajectories.

The RC trajectories for 8-site *P. aestuarii* and 7-site *C. tepidum* FMO complexes are shown in Figure 9. This is the first time we have seen the time evolution of the nuclear reaction coordinate in the EET dynamics of the FMO complex. The similarities between the 8-site *P. aestuarii* and 7-site *C. tepidum* FMO complexes are seen, but the differences are more interesting to note. For instance, the RC component *y*_1_ that connects the PES minima of |1⟩ and |2⟩ displays a larger oscillation in the 7-site *C. tepidum* FMO, which is consistent with the larger oscillation in the |1⟩ and |2⟩ population dynamics in Figure 7 than the 8-site *P. aestuarii* case in Figure 8. Moreover, there are typically two time scales reflected by the RC dynamics. The first ultrafast time scale is about 500 fs, when all RC components respond to the photoexcitation quickly. The second time scale is about a couple of picoseconds, during which the RC components *y*_2_, *y*_3_, *y*_4_, *y*_6_ in both FMO complexes and the additional *y*_7_ in the *P. aestuarii* FMO complex continue to drift, contributing to the longer EET dynamics that last several picoseconds. We note that the amplitudes of the RC components in the 7-site *C. tepidum* FMO complex are about half of those in the 8-site *P. aestuarii* FMO complex, which can be traced back to the reaction coordinate frequencies of 1187.2 and 507.68 cm^–1^, respectively. The different RC dynamics in these components indicate a signature of heterogeneity in different BChl sites, along with their protein scaffold environments.

In fact, heterogeneous environment is crucial in shaping the nonadiabatic dynamics and it is more significant in the photoinduced CT process than in the EET process,^53^ which is due to the fact that the CT state interacts with the surrounding environment quite differently from the LE state, thus a large reorganization energy would be expected. Accordingly, we believe that it is important to account for the reorganization energies between all pairs of states in studying CT dynamics in a heterogeneous environment. Moreover, even in the case when all chromophores are identical—neglecting molecular orientations and shapes—and they are buried in an identical solvent environment, we would still expect heterogeneous reorganization energies. This is because the intermolecular distances cannot be the same for more than four molecules in our usual three-dimensional space, i.e., four molecules locate at vertices of a regular tetrahedron, and adding one more molecule would introduce a new pair distance, which will break the equal-distance symmetry. Thus, the treatment for heterogeneous environments is necessary for constructing consistent effective models for multistate systems, such as the approach introduced in parametrizing the MSH or MRC models.

**Figure 10:**
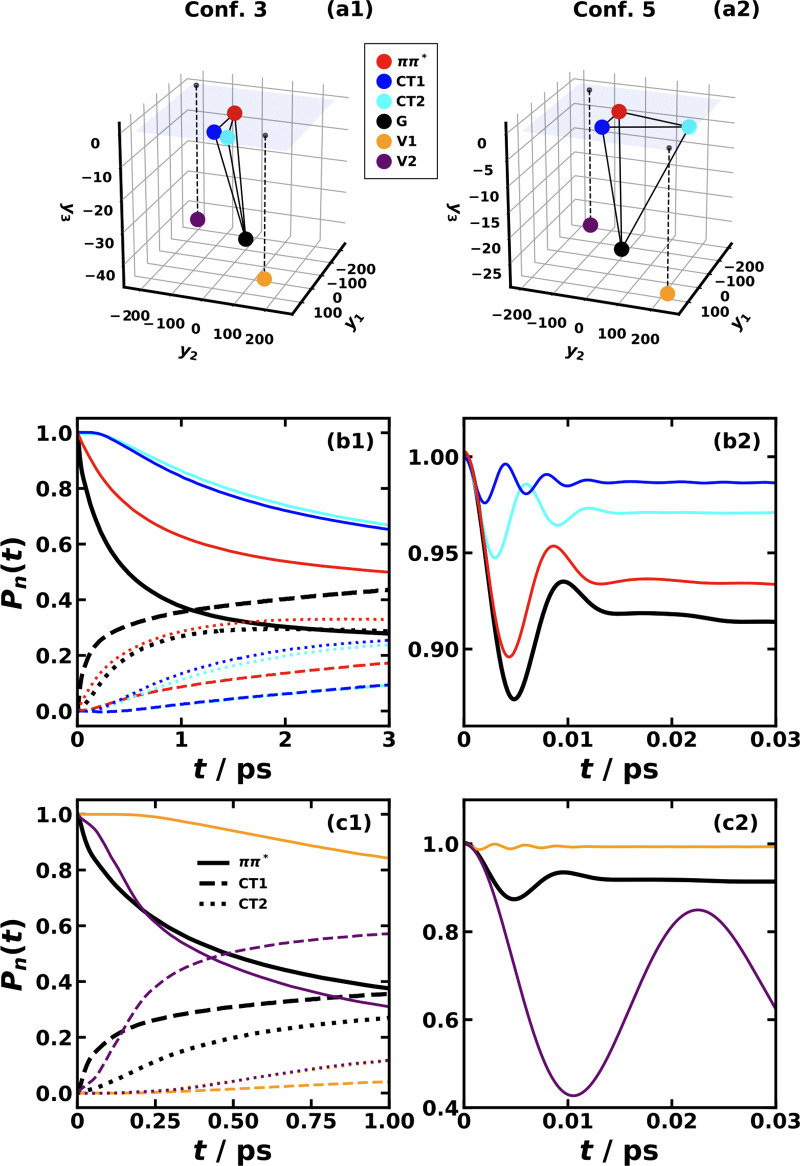
Comparison of nonequilibrium nuclear initial shifts (in a.u.) using MRC models of CPC_60_ triad conformations #3 (left) and #5 (right) indicated as circles in 3-dimensional RC space (a1) and (a2), respectively. The nonadiabatic dynamics are obtained with SQC method at 300 K. The initial electronic state is |ππ*⟩⟨ππ*| in all cases. Panels (b1) and (b2) are cases with the initial nuclear state starting from ππ* (red), CT1 (blue), and CT2 (cyan) states, and panels (c1) and (c2) are cases with the initial nuclear state starting from arbitrarily chosen V1 (orange) and V2 (purple) states compared with the original case starting from the ground (G, black) state.

Finally, we would like to show another advantage of the MRC model, i.e., the straightforwardness in starting from an arbitrary nuclear initial state determined by the RC components.^78,92^ Taking Figure 10 for the triad system as an example, we visualize the tetrahedron formed by the PES minima of the 4 electronic states in the RC space in 3-dimensions including ππ*, CT1, CT2, and G states, as well as two arbitrary states called V1 and V2 (along with their projections to the upper plain). Note that the initial electronic state is |ππ*⟩⟨ππ*| in all cases, and we only change the initial nuclear state. It is clear that the PES minima distances in conf. #3 and conf. #5 are different; especially, CT1 and CT2 are closer in conf. #3 than in conf. #5 leading to a significant CT2 population in the first couple of picoseconds in conf. #3 (see black dots in Figure 10(b1)). If the initial nuclear state starts from other excited states such as ππ*, CT1, and CT2, the population transfer is actually slower than starting from the ground state as seen in Figure 10(b1,b2) for both conformations. To see the trend of transfer rate enhancement or suppression, we consider the cases starting from two extreme states, i.e., V1 and V2, as shown in Figure 10(c1,c2), and discover that more negative *y*_1_, *y*_2_ values such as the purple V2 lead to faster population transfer, and more positive *y*_1_, *y*_2_ values such as the orange V1 lead to slower population transfer, which is also consistent with the above cases starting from the excited states. The results may shed light on the rational design of more efficient light-harvesting systems by tuning the nuclear distribution.

## 5 Concluding Remarks

In this work, we proposed a systematic way to construct MRC model Hamiltonian from all-atom simulations for understanding charge and energy transfer dynamics in complex condensed phases. We do so by first proving the equivalence between the MRC model and the MSH model,^53^ which has been shown to consistently account for the heterogeneous correlations between all pairs of states by extending the spatial dimensions to *F* – 1 for each normal mode in an *F*-state system and MSH agrees with all-atom nonadiabatic semiclassical dynamics.^54^ The reaction coordinate in an *F*-state MRC model also has *F* – 1 dimensions. The distances between the PES minima of all pairs of states are selected such that the reorganization energies are carried by the reaction coordinate in the extended space, and in the meanwhile, the reaction coordinate bilinearly couples to the secondary bath. In our numerical simulations for the photoinduced CT dynamics in the CPC_60_ triad in the solution phase as well as the EET dynamics in a photosynthetic FMO complex using several nonadiabatic dynamical methods, we confirm that the MRC and the MSH models give rise to the same nonadiabatic dynamics using the same semiclassical dynamical methods, thus demonstrating their equivalence. Besides, MRC model offers an intuitive physical picture for the nuclear-electronic feedback in nonadiabatic processes, such as the inherent average trajectory and the characteristic frequency of the reaction coordinate as well as a straightforward way to incorporate nonequilibrium nuclear initial conditions. Because of these features including mapping from all-atom information, consistently incorporating multistate interstate correlations or reorganization energies, intuitive physical picture using the reaction coordinate in extended dimensions, and nonequilibrium initial state, the MRC model Hamiltonian is believed to provide an effective and robust platform for quantum dissipative dynamics in complex condensed-phase systems. Both MSH and MRC models are based on the harmonic assumption that is expected to be valid for condensed-phase systems, where Gaussian statistics is supported by the central limit theorem and the agreement between MSH model and all-atom nonadiabatic dynamics of the triad.^54^ In contrast, the MSH or MRC models are not expected to be accurate in certain single-molecule systems, where anharmonic modes such as isomerization dihedrals might be important. Many quantum dynamical formalisms such as the generalized quantum master equation (GQME)^93−95^ are based on the system–bath type of Hamiltonian, and the MRC model can be written in such a way, so MRC model could be naturally adopted for answering the questions regarding realistic systems using such approaches. MRC model could also describe linear vibronic couplings, which will be important for nonadiabatic transitions in conical intersections.^63−65,92,96^ Work on using MRC models to investigate the effects of realistic spectral density, state-dependent system–bath couplings, reaction pathways, and heterogeneous environments due to static and dynamical disorder is underway and will be reported in future publications.^3,9,97,98^

## A. Equilibrium Shifts in MSH and MRC Models

As shown in ref (53), the equilibrium shifts in MSH model are proportional to the coordinates of polyhedron vertices

64

and as described in the main text, the equilibrium shifts of the reaction coordinate in MRC model are given by

65

Here, the *F* polyhedron vertices are chosen such that the distances between the PES minima of *F*(*F* – 1)/2 pairs of states are proportional to the corresponding square root of the reorganization energies, . The positions of the *F* polyhedron vertices are given by

66

67

68

69

70

71

where

72

73

and more generally for (4 ≤ *j* < *k*):

74

## Supporting Information Available

See the Supporting Information for the . The Supporting Information is available free of charge at https://pubs.acs.org/doi/10.1021/acs.jctc.3c00770.

MRC parameters (ZIP)
MRC model parameters for CPC_60_ triad conformations #3 and #5, and *C. tepidum* and *P. aestuarii* FMO complexes (PDF)
