# Supplementary material for: Multistate Reaction Coordinate Model for Charge and Energy Transfer Dynamics in the Condensed Phase
Source: J Chem Theory Comput. 2023 Oct 10;19(20):7151–70. doi: 10.1021/acs.jctc.3c00770 (PMC10601487; doi:10.1021/acs.jctc.3c00770)
Supplement: Supplementary file 2 — ct3c00770_si_002.pdf [file ct3c00770_si_002.pdf]

Supporting Information: Multi-State Reaction Coordinate Model for Charge and Energy Transfer Dynamics in the Condensed Phase

Zengkui Liu,[†,‡,¶] Haorui Hu,[†] and Xiang Sun[*,†,‡,§,¶]

†*Division of Arts and Sciences, NYU Shanghai, 567 West Yangsi Road, Shanghai 200124, China*

‡*NYU-ECNU Center for Computational Chemistry at NYU Shanghai, 3663 Zhongshan Road North, Shanghai 200062, China*

¶*Department of Chemistry, New York University, New York, New York 10003, United States*

§*Shanghai Frontiers Science Center of Artificial Intelligence and Deep Learning, NYU Shanghai, 567 West Yangsi Road, Shanghai, 200124, China*

E-mail: xiang.sun@nyu.edu

1 MRC Model Parameters

We summarize the Multi-state Reaction Coordinate (MRC) model parameters for the carotenoid-porphyrin-C_{60} (CPC_{60}) triad conformations #3 and #5 with $F = 4$ states, as well as the photosynthetic light-harvesting Fenna-Matthews-Olson (FMO) complex in *C. tepidum* with 7 BChl sites ($F = 8$ states including the ground state) and FMO complex in *P. aestuarii* with 8 BChl sites ($F = 9$ states including the ground state). We list the parameters of the reaction coordinate (RC) here, and provide the additional secondary bath parameters in the attached files, respectively:

1. MRC_param_Conf3.txt
2. MRC_param_Conf5.txt
3. MRC_param_C_FMO.txt
4. MRC_param_P_FMO.txt

The RC equilibrium shift matrices of triad Conf. #3 and Conf. #5 are given in the main text in Eqs. 55 and 56. The diagonal and off-diagonal system Hamiltonian matrix elements (in eV) including the vertical energy shifts $\{\varepsilon_X\}$ and the diabatic couplings $\{\Gamma_{XY}|X \neq Y\}$ of Conf. #3 and Conf. #5 are respectively

$$\mathbf{H}_s = \begin{pmatrix} 0 & -0.01467393 & 0.007201061 & 0 \\ -0.01467393 & -0.850223 & -0.02924042 & 0 \\ 0.007201061 & -0.02924042 & -0.665904 & 0 \\ 0 & 0 & 0 & 0 \end{pmatrix} \tag{S1}$$

and

$$\mathbf{H}_s = \begin{pmatrix} 0 & 0.0810422 & 0.004101633 & 0 \\ 0.0810422 & -0.75840926 & -0.003242261 & 0 \\ 0.004101633 & -0.003242261 & -1.12789614 & 0 \\ 0 & 0 & 0 & 0 \end{pmatrix}. \tag{S2}$$

For the FMO complex, the RC equilibrium shift matrices (in a.u.) for 7-site *C. tepidum* FMO

and 8-site *P. aestuarii* FMO are respectively

$$\mathbf{S} = \begin{pmatrix} 5.367560 & 0 & 0 & 0 & 0 & 0 & 0 \\ 2.073971 & 4.962300 & 0 & 0 & 0 & 0 & 0 \\ 1.985571 & 1.390814 & 4.133696 & 0 & 0 & 0 & 0 \\ 2.024756 & 1.373960 & 1.218606 & 4.632110 & 0 & 0 & 0 \\ 2.044373 & 1.375045 & 1.169529 & 0.807617 & 3.927971 & 0 & 0 \\ 2.023285 & 1.325252 & 1.123716 & 0.743360 & 0.738099 & 4.320583 & 0 \\ 2.023268 & 1.339293 & 1.180830 & 0.754466 & 0.746868 & 0.551406 & 1.442052 \end{pmatrix} \tag{S3}$$

and

$$\mathbf{S} = \begin{pmatrix} 13.06593 & 0 & 0 & 0 & 0 & 0 & 0 & 0 \\ 4.409819 & 11.77731 & 0 & 0 & 0 & 0 & 0 & 0 \\ 4.363315 & 3.257221 & 9.699128 & 0 & 0 & 0 & 0 & 0 \\ 4.403633 & 3.195909 & 2.884700 & 11.96838 & 0 & 0 & 0 & 0 \\ 4.469611 & 3.295033 & 2.753279 & 1.443972 & 9.238980 & 0 & 0 & 0 \\ 4.438910 & 3.356138 & 2.838160 & 1.667363 & 1.826184 & 10.23264 & 0 & 0 \\ 4.485492 & 3.286568 & 2.775246 & 1.541134 & 1.902571 & 1.106586 & 9.401581 & 0 \\ 4.488708 & 3.277690 & 2.882332 & 1.533064 & 1.799663 & 1.301859 & 1.289319 & 3.209442 \end{pmatrix}. \tag{S4}$$

The diagonal and off-diagonal system Hamiltonian matrix elements (in cm^{-1}) including the vertical energy shifts $\{\varepsilon_X\}$ and the diabatic couplings $\{\Gamma_{XY}|X \neq Y\}$ of 7-site *C. tepidum* FMO

complex are adopted from Ref. 1:

$$\mathbf{H}_s = \begin{pmatrix} 12410 & -87.7 & 5.5 & -5.9 & 6.7 & -13.7 & -9.9 & 0 \\ -87.7 & 12530 & 30.8 & 8.2 & 0.7 & 11.8 & 4.3 & 0 \\ 5.5 & 30.8 & 12210 & -53.5 & -2.2 & -9.6 & 6 & 0 \\ -5.9 & 8.2 & -53.5 & 12320 & -70.7 & -17 & -63.3 & 0 \\ 6.7 & 0.7 & -2.2 & -70.7 & 12480 & 81.1 & -1.3 & 0 \\ -13.7 & 11.8 & -9.6 & -17 & 81.1 & 12630 & 39.7 & 0 \\ -9.9 & 4.3 & 6 & -63.3 & -1.3 & 39.7 & 12440 & 0 \\ 0 & 0 & 0 & 0 & 0 & 0 & 0 & 0 \end{pmatrix}. \tag{S5}$$

The diagonal and off-diagonal system Hamiltonian matrix elements (in cm^{-1}) including the vertical energy shifts $\{\varepsilon_X\}$ and the diabatic couplings $\{\Gamma_{XY}|X \neq Y\}$ of 8-site *P. aestuarii* FMO complex are adopted from Ref. 2 :

$$\mathbf{H}_s = \begin{pmatrix} 310 & -97.9 & 5.5 & -5.8 & 6.7 & -12.1 & -10.3 & 37.5 & 0 \\ -97.9 & 230 & 30.1 & 7.3 & 2.0 & 11.5 & 4.8 & 7.9 & 0 \\ 5.5 & 30.1 & 0 & -58.8 & -1.5 & -9.6 & 4.7 & 1.5 & 0 \\ -5.8 & 7.3 & -58.8 & 180 & -64.9 & -17.4 & -64.4 & -1.7 & 0 \\ 6.7 & 2.0 & -1.5 & -64.9 & 405 & 89.0 & -6.4 & 4.5 & 0 \\ -12.1 & 11.5 & -9.6 & -17.4 & 89.0 & 320 & 31.7 & -9.7 & 0 \\ -10.3 & 4.8 & 4.7 & -64.4 & -6.4 & 31.7 & 270 & -11.4 & 0 \\ 37.5 & 7.9 & 1.5 & -1.7 & 4.5 & -9.7 & -11.4 & 505 & 0 \\ 0 & 0 & 0 & 0 & 0 & 0 & 0 & 0 & 0 \end{pmatrix}. \tag{S6}$$

2 Nonequilibrium initial nuclear state for triad

In Fig. 10 of the main text, we plot the different nonequilibrium initial nuclear states for the triad system, where for better visualization, we flip the z-axis making the ground state below the plane

formed by $\pi\pi^*$, CT1, and CT2 states' PES minima. If we project all the states' PES minima to this plane, we have 2-dimensional projection as shown in Fig. S1. The two arbitrarily chosen virtual states, i.e., V1 and V2 are different in Conf. #3 and Conf. #5. The RC shift components in the V1 and V2 states of Conf. #3 are given by (in a.u.)

$$\mathbf{s}_{V1} = (-203.53037877850758, -203.53037877850758, 43.70973123755616), \quad (S7)$$

$$\mathbf{s}_{V2} = (150, 150, 43.70973123755616). \quad (S8)$$

The RC shift components in the V1 and V2 states of Conf. #5 are given by (in a.u.)

$$\mathbf{s}_{V1} = (-150, -150, 27.41264226809738), \quad (S9)$$

$$\mathbf{s}_{V2} = (230.56798696652558, 230.56798696652558, 27.41264226809738). \quad (S10)$$

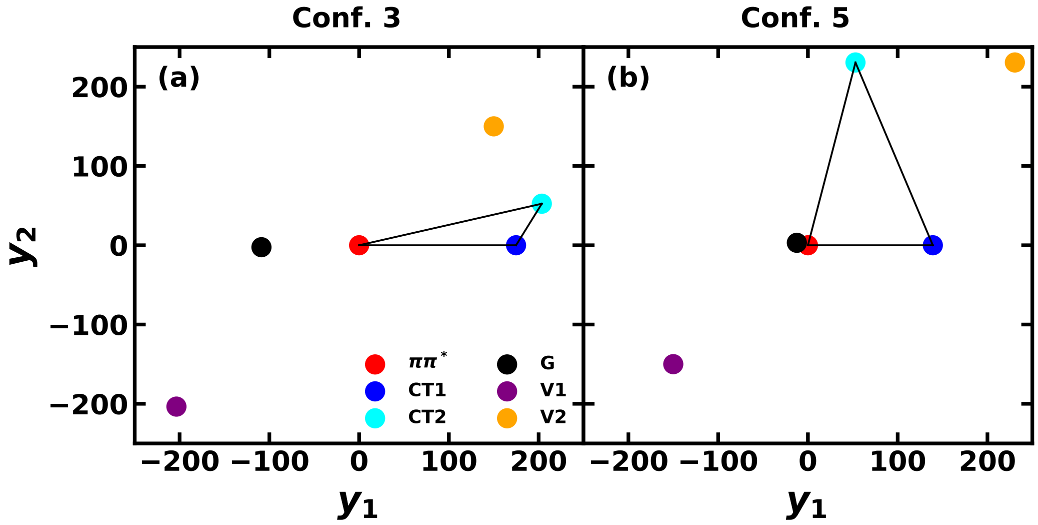

Figure S1: The reaction coordinate (RC) shifts (in a.u.) of different states in triad conformations #3 and #5 projected to the $y_3 = 0$ plane.

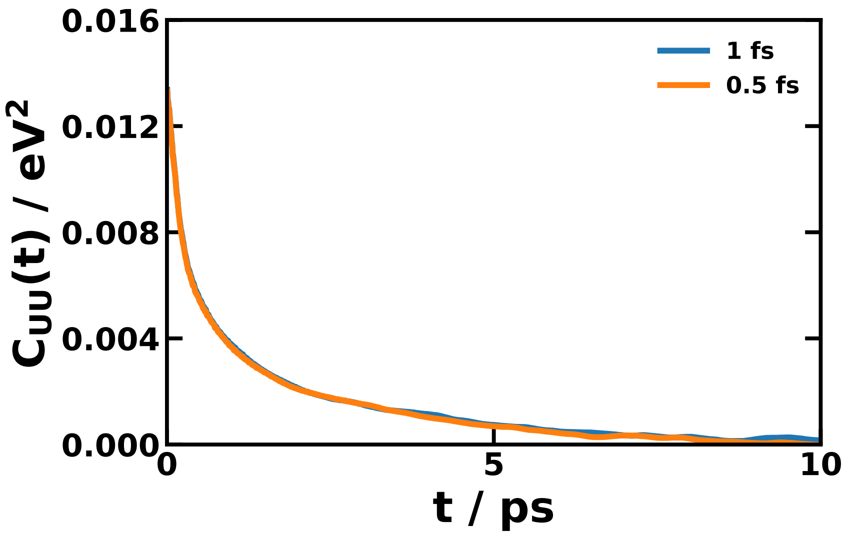

Figure S2: Comparison of the energy gap time correlation function for triad conformation #5 and the energy gap is between $\pi\pi^*$ and CT1 sampled on $\pi\pi^*$ state, obtained with time step δt =1 fs and 0.5 fs.

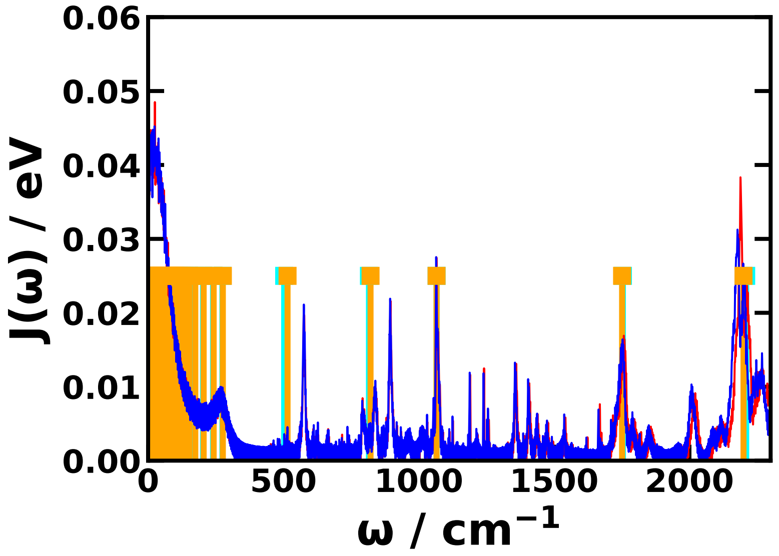

Figure S3: Comparison of the spectral densities and discretized normal mode frequencies for triad conformation #5 obtained with time step δt =1 fs (red for $J(\omega)$ and cyan lines for frequencies) and 0.5 fs (blue for $J(\omega)$ and orange lines for frequencies).

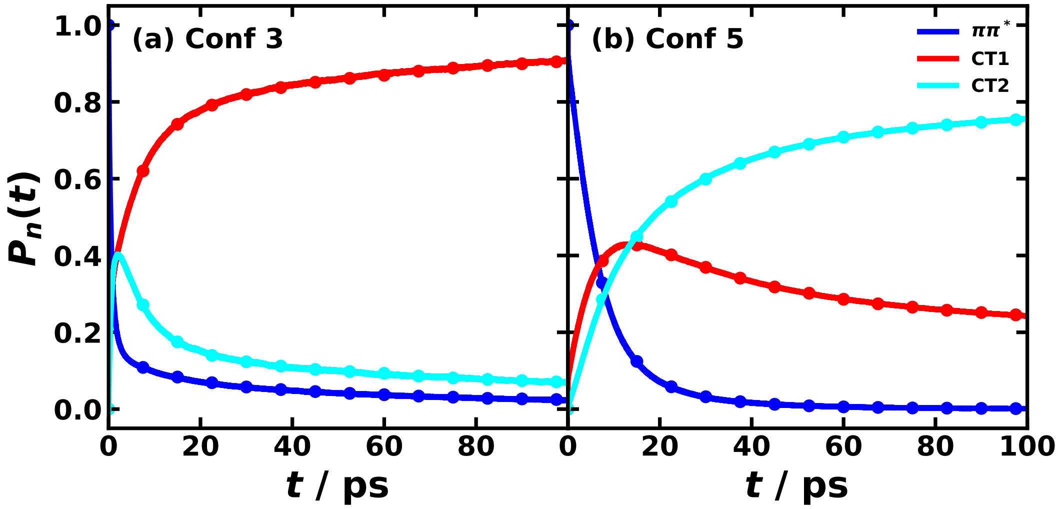

Figure S4: Long-time nonadiabatic dynamics (100 ps) of triad conformations #3 and #5 at 300 K obtained with MRC (dotted) and MSH (line) models and SQC dynamical method.

References

(1) Adolphs, J.; Renger, T. How Proteins Trigger Excitation Energy Transfer in the FMO Complex of Green Sulfur Bacteria. *Biophys. J.* **2006**, *91*, 2778–2797.

(2) Moix, J.; Wu, J.; Huo, P.; Coker, D.; Cao, J. Efficient Energy Transfer in Light-Harvesting Systems, III: The Influence of the Eighth Bacteriochlorophyll on the Dynamics and Efficiency in FMO. *J. Phys. Chem. Lett.* **2011**, *2*, 3045–3052.
